# Cardiovascular-CNS Comorbidity as a Means to Rationalize Serendipity in Drug Discovery and Advance Translational Research

**DOI:** 10.1155/2010/318167

**Published:** 2010-01-19

**Authors:** Hari Manev

**Affiliations:** Department of Psychiatry, The Psychiatric Institute, University of Illinois at Chicago, 1601 West Taylor Street, MC912, Chicago, IL 60612, USA

It has been argued that “lamp-post research” is the main culprit in the lack of major breakthroughs in neuropsychiatric drug discovery. An old joke goes like this. A drunk loses his keys and looks for them under a lamp-post. A passerby asks what he is doing. He answers: “Looking for my keys that I lost in a dark alley two blocks away.” “Then why are you looking for them under this lamp-post?” wonders the passerby. “Because I can see much better here.” Not all researchers looking for novel neuropsychiatric pharmacological treatments are all drunks, but the alternative to lamp-post research (which mostly generates “me-too” drugs rather than entirely new compounds) appears to be even less rational—it typically involves serendipity.

Several strategies have been proposed to overcome reliance on pure serendipity, which occurs too rarely to be counted on as a consistent source of drug discovery. These strategies include cell-phenotype-based and organism-phenotype-based approaches to the rationalization of serendipity for drug discovery. Phenotype-based approaches to drug discovery rely on the notion that putative therapeutic molecules can be discovered in the absence of any knowledge about a disease mechanism (i.e., molecular target) if these molecules are capable of reversing a disease phenotype. A historical example of serendipitous organism-phenotype-based approach is the discovery of the first antidepressant agents. Briefly, in the search for a tuberculostatic at the end of World War II, stocks of the leftover rocket propellant hydrazine were used to produce its chemical derivatives, isoniazid and iproniazid, which were found to be potently tuberculostatic. Physicians who more than 60 years ago used these drugs for treatment of tuberculosis observed that, in addition to the healing of tubercular lesions, isoniazid and even more so, iproniazid, produced favorable mental “side effects.” This serendipitous organism-phenotype-based discovery led to the testing of iproniazid in depressed patients and finding its antidepressant action.

Recent indications of putative mechanisms responsible for the cooccurrence of cardiovascular and psychiatric/neurological disorders point to an additional possibility for improving the process of serendipity rationalization in drug discovery—by focusing on the cooccurrence of these disorders.

As an example, one could focus on the cooccurrence of major depression (MD) and coronary heart disease (CHD), in which depression is independently associated with increased cardiovascular morbidity and mortality. Serendipity could be rationalized by specifically targeting patients with cooccurring MD and CHD (or alternatively using animal models of this cooccurrence) and applying the CHD-phenotype-based approach in evaluating drugs (standard or novel/experimental) administered primarily for the treatment of the MD component of the cooccurrence. A successful screen performed in this manner could lead to the discovery of novel families of cardiovascular drugs. Alternatively, one could use the MD-phenotype-based approach in evaluating drugs for the treatment of the CHD component of this cooccurrence to find novel antidepressants. For such an approach to be successful, no initial knowledge is required about the putative mechanistic links between MD and CHD. Once effective drugs/molecules are identified, this knowledge could be used to elucidate the participating biological/molecular mechanisms. Similar approaches could be developed for other cooccurring cardiovascular and psychiatric/neurological disorders.

The current establishment of a new network of NIH- (National Institutes of Nealth-) supported Clinical and Translational Research Centers (CTSCs) in the USA emphasizes a renewed interest in fostering closer ties between basic and clinical research. If these ties succeed in rationalizing serendipity in the search for novel neuropsychiatric and cardiovascular pharmacological treatments, the new CTSCs could prove to be a way out of discredited lamp-post research and also a way to stop relying on the serendipitous discoveries that have proved so scarce in the past. *Cardiovascular Psychiatry and Neurology* looks forward to the results of these future studies.

*Hari Manev*

